# Vitamin D levels and Vitamin D-related gene polymorphisms in Chinese children with type 1 diabetes

**DOI:** 10.3389/fped.2022.965296

**Published:** 2022-10-05

**Authors:** Xiaofang Chen, Jia Fu, Ying Qian, Xiufang Zhi, Linjie Pu, Chunyu Gu, Jianbo Shu, Ling Lv, Chunquan Cai

**Affiliations:** ^1^ Tianjin Pediatric Research Institute, Tianjin Children's Hospital (Children's Hospital of Tianjin University), Tianjin, China; ^2^ Graduate College of Tianjin Medical University, Tianjin Medical University, Tianjin, China; ^3^ Tianjin Pediatric Research Institute, Tianjin Children's Hospital (Tianjin University Children's Hospital), Tianjin, China; ^4^ Department of Endocrinology, Tianjin Children's Hospital (Tianjin University Children's Hospital), Tianjin, China; ^5^ Tianjin Key Laboratory of Birth Defects for Prevention and Treatment, Tianjin Children's Hospital (Children's Hospital of Tianjin University), Tianjin, China

**Keywords:** CYP2R1, polymorphism, vitamin D, T1D, Chinese children, GMDR

## Abstract

Low vitamin D levels may play a role in type 1 diabetes (T1D) susceptibility. Since 25(OH)D synthesis is genetically regulated, single nucleotide polymorphisms (SNPs) of important genes have also been shown to modulate the risk of T1D, so this study aimed to investigate the relationship between five SNPs in CYP2R1, DHCR7, CYP24A1, VDR genes, serum 25(OH)D levels and T1D in Chinese children. This case-control study included 141 T1D patients and 200 age-matched healthy children.25 (OH) D concentration was determined, genotyping was performed by High resolution melting (HRM). There was a significant difference in the prevalence of vitamin D deficiency, insufficiency, and sufficiency between T1D and healthy controls. (*χ*^2^ = 10.86, *p* = 0.004), however no evidence of the association between any group of SNPs and circulating 25(OH) D levels was observed. The allele distribution of CYP2R1(rs1993116) was significantly different between T1D and control group (*p* = 0.040), and the C allele carriers of rs1993116 had a higher risk of T1D than the T allele carriers, Carriers of the CC and CT genotypes of rs1993116 have higher T1D risk than those carrying the TT genotype. GMDR analysis revealed a significant interaction between CYP2R1(rs12794714) and CYP2R1(rs1993116) in the risk of T1D with a maximum testing balance accuracy of 60.39%.

## Introduction

Type 1 diabetes (T1D) is generally considered a chronic autoimmune disease caused by the destruction of insulin-producing pancreatic beta cells (1, 2), although T1D can occur at any age, it is one of the most common chronic diseases in childhood. Peak clinical presentation occurs between 5 and 7 years of age, at or near adolescence (3, 4). Globally, both the incidence and prevalence of T1D are increasing, however the greatest increase in incidence occurs in children under 15 years of age, especially in children under 5 years of age (5), These increases cannot be fully explained by genetic changes, involving environmental or behavioral factors, or both. T1D, once considered a single autoimmune disease, is now increasingly recognized as caused by complex interactions between environmental factors and the microbiome, genome, metabolism, and immune system (6). Many environmental factors have been associated with T1D, including diet, vitamin D deficiency, viruses associated with islet inflammation (e.g., enteroviruses), and decreased gut—microbiome diversity. There is growing evidence that low vitamin D concentrations are strongly associated with a variety of adverse outcomes, such as asthma, diabetes, cardiovascular disease, and certain cancers (7–10). Common genetic variants affecting circulating 25(OH) D levels may be important in enhancing our understanding of the observed associations between vitamin D status and several diseases.

Vitamin D is a fat-soluble steroid, ergocalciferol (vitamin D2) is produced by ultraviolet irradiation of the plant sterol ergosterol, and cholecalciferol (vitamin D3) is produced from its precursor 7-dehydrocholesterol (7-DHC) synthesized in human skin under ultraviolet light. Epidemiological evidence suggests that vitamin D deficiency is associated with the pathogenesis of T1D (11, 12). as 25(OH) D synthesis is genetically regulated, single nucleotide polymorphisms (SNPs) may alter bioavailability and targeting effects of vitamin D metabolites, polymorphisms in genes critical for vitamin D metabolism have also been shown to modulate T1D risk (13). Genome-wide association studies(GWAS) have confirmed that distinct polymorphisms in genes involved in vitamin D metabolism may affect islet autoimmunity and risk of T1D, SNPs of important genes involved in synthesis (7-dehydrocholesterol reductase, *DHCR7),* hydroxylation (cytochrome P450 family 2 subfamily R member 1, *CYP2R1*), degradation (cytochrome P450 family 24 subfamily A member 1, *CYP24A1*) and transcription (vitamin D receptor, *VDR*) (14, 15). The purpose of this study was to evaluate the relationship between *CYP2R1*(rs1993116, rs12794714), *DHCR7*(rs12785878), *CYP24A1* (rs17216707), *VDR* (rs1544410), T1D and vitamin D levels in Chinese children.

## Materials and methods

### Subjects

This study was designed as a retrospective case-control study, with all subjects from Tianjin Children's Hospital. The case group included 141 patients with T1D (59 males and 82 females), the mean age at the time of study ± standard deviation (SD) = (6.3 ± 3.3 years), Diagnosis of T1D is based on typical clinical presentation, high serum HbA1c level, low serum C-peptide level, and presence of one or more pancreatic autoantibodies (16). The control group included 200 healthy children (114 males and 86 females, mean age ± SD = (5.6 ± 3.6 years). The study was approved by the Medical Ethics Committee of Tianjin Children's Hospital.

### Detection of serum vitamin D levels

Serum samples were collected during 2017–2019, and stored frozen at −80 °C until analysis. Serum 25 (OH) D2 and 25 (OH) D3 levels were detected by liquid chromatography-tandem mass spectrometry (LC-MS/MS), and the sum of the two was 25 (OH) D concentration. To compare the prevalence of vitamin D deficiency in T1D and controls, 25 (OH) D levels were divided into deficiency (<20 ng/ml), insufficient (20–29 ng/ml), and sufficient (≥30 ng/ml) (17).

### Genotyping

High-resolution melting (HRM) analysis has been tested in a variety of clinical mutation-scanning and genotyping applications and proved to be sensitive (18), so we used HRM for genotyping in this study. Genomic DNA was extracted from whole blood using the Genomic blood DNA mini kit (Beijing ComWin Biotech.) according to the manufacturer's protocol, and stored at −20 °C for later use. According to the website (https://www.ncbi.nlm.nih.gov) and previous research, Primers were designed using DNAMAN9 software and PCR amplification was performed. The amplified products obtained were sent to Sanger sequencing. The sequencing results were analyzed with Chromas2.6.4 sequence analysis software and the required High-resolution melting reference samples were found. Amplification was performed in a 20 *μ*l reaction volume containing 10 *μ*l Forget-Me-Not^TM^ EvaGreen^Ⓡ^qPCR Master Mix (Biotum, USA), 8 *μ*l ddH20, 0.5 *μ*l forward primer, 0.5 *μ*l reverse primer, DNA 1 *μ*l. HRM was performed using a LightCycler^Ⓡ^480, 1 min at 95 °C, 1 min at 40 °C, melting was done from 55 °C to 98 °C at 0.1 °C/sec, melting curves were analyzed with LightCycler 480 Software 1.5, After data collection, melting curves are normalized by selecting a linear region before and after the melting transition.

### Statistical analysis

SPSS software (version 26.0) was used for the data analysis. Mann-Whitney U test was used to detect the association between SNPs genotype and serum 25(OH)D level. The Chi-square test was used to calculate genotype and allele frequencies in cases and controls, and assessment of Hardy-Weinberg equilibrium (HWE) for controls. Logistic regression adjustment for covariates (age, sex) was performed to investigate the association between five SNPs in the *CYP2R1*, *DHCR7, CYP24A1* genes, and T1D risk. Analysis of SNP-SNP interactions associated with T1D risk using generalized multifactorial dimensionality reduction (GMDR, version 0.7, University of Virginia, USA). Two-tailed *P*-values <0.05 were considered statistically significant for all tests.

## Results

### Clinical and biochemical data and prevalence of vitamin D deficiency in T1d

Baseline characteristics of the study population and the prevalence of 25 (OH) D deficiency in children with T1D and healthy controls are shown in Table 1. There was no significant difference in mean age at the time of the case and control studies, (*χ*^2^ = 1.74, *p *= 0.083), but there was a significant difference in the ratio of males and females.(*χ*^2^ = 7.6, *p *= 0.006). For the T1D group, 56.0% were vitamin D deficient, 31.9% were vitamin D Insufficiency, and 12.1% were vitamin D Sufficiency, while the control group 38.5% were vitamin D deficient, 48.0% were vitamin D Insufficiency, and 13.5% were vitamin D Sufficiency. There was a significant difference in the prevalence of vitamin D deficiency, insufficiency, and sufficiency between T1D and healthy controls. (*χ*^2^ = 10.86, *p *= 0.004). Vitamin D deficiency are more prevalent in T1D.

**Table 1:** Characteristics of the study population.

Characteristics	T1D (*n* = 141)	Control (*n *= 200)	*t/Z/χ^2^*	*p*
Age, years (mean ± SD)	6.3 ± 3.3	5.6 ± 3.6	1.74	0.083
Gender				
Male	59 (41.8%)	114 (57.0%)	7.6	0.006
Female	82 (58.2%)	86 (43.0%)		
Duration of T1DM (months)	6.0 (2.0–24.0)			
TC (mmol/l)	5.0 ± 1.4			
TG (mmol/l)	3.1 ± 3.7			
HDL-C (mmol/l)	1.4 ± 0.5			
LDL-C (mmol/l)	2.9 ± 1.2			
ALP, (IU/l)	266.9 ± 99.6			
Vit D status				
deficiency (≤20 ng/ml)	79 (56.0%)	77 (38.5%)		
Insufficiency (20–30 ng/ml)	45 (31.9%)	96 (48.0%)	10.86	0.004
sufficiency (≥30 ng/ml)	17 (12.1%)	27 (13.5%)		

TC, total cholesterol; TG, triglyceride; HDL, high-density lipoprotein cholesterol; LDL, low-density lipoprotein cholesterol.

ALP, Alkaline phosphatase.

### Genotypes and serum 25(Oh) D levels

The relationship between the genotypes of the five SNPs and serum 25 (OH) D levels in patients and controls is shown in Table 2. The relationship between SNPs genotypes and 3 groups of vitamin D levels (deficient, insufficient and sufficient) is shown in the supplementary materials section (Supplementary Table S1). In the control group, *CYP2R1* (rs12794714) not complied with Hardy-Weinberg equilibrium, there may be selection bias. Deviation from Hardy-Weinberg equilibrium was not observed in the other four SNPs, the chi-square test is shown in the supplementary materials section (Supplementary Table S2). However, no evidence of association between any group of SNPs and circulating 25 (OH) D levels was observed in our study.

**Table 2:** Relationship between SNPs genotypes and serum 25(OH) D levels.

SNPs	25 (OH)D (ng/ml)			*χ*2	*P*
	GG	TG	TT		
DHCR7 (rs12785878)					
Total	21.40 (14.13,24.99)	20.25 (14.16–23.12)	20.92 (14.57–25.39)	0.884	0.643
Case	21.05 (15.1–31.57)	17.77 (13.07–24.35)	19.13 (14.08–25.35)	3.620	0.164
Control	21.40 (13.00–22.47)	21.40 (15.23–22.80)	21.40 (16.47–26.87)	0.764	0.683
	CC	CT	TT		
CYP2R1 (rs12794714)					
Total	21.40 (14.53–25.68)	20.12 (12.95–24.26)	20.48 (15.02–22.70)	2.252	0.324
Case	19.12 (14.20–25.60)	19.25 (12.84–25.37)	18.17 (14.39–25.36)	0.554	0.758
Control	21.39 (15.34–26.24)	21.40 (12.83–23.63)	21.40 (15.90–22.55)	1.503	0.472
	CC	CT	TT	CC	
CYP2R1 (rs1993116)					
Total	20.41 (14.94–24.59)	20.70 (13.69–24.67)	21.40 (14.12–26.55)	0.112	0.945
Case	18.75 (14.35–25.96)	18.50 (13.30–25.01)	21.82 (14.31–27.30)	0.532	0.767
Control	21.40 (15.52–23.62)	21.40 (15.85–23.74)	20.58 (14.10–26.27)	0.242	0.886
	TT	CT			
CYP24A1 (rs17216707)					
Total	20.95 (14.38–24.69)	18.76 (14.11–23.89)		0.828	0.363
Case	19.02 (13.58–25.36)	18.76 (14.50–27.30)		0.153	0.696
Control	21.40 (15.17–24.28)	18.79 (12.25–21.40)		2.762	0.096
	GG	GA			
VDR (rs1544410)					
Total	20.59 (14.23–24.69)	21.40 (15.68–24.19)		0.241	0.623
Case	18.75 (13.66–25.19)	23.29 (15.13–27.97)		0.846	0.358
Control	21.40 (14.70–24.46)	21.31 (17.44–22.33)		0.177	0.674

The distribution of continuous variable serum 25(OH)D is skewed, then Mann-Whitney U was applied to test the relationship between SNPs genotype and 25(OH)D levels, described as interquartile range P50 (P25, P75).

### Genotype and T1d risk

The genotypes and allele frequencies of *CYP2R1*, *DHCR7*, *CYP24A1,* and *VDR* observed in patients and controls are shown in Table 3. The allele distribution of *CYP2R1* (rs1993116) was significantly different between T1D and control group (*p* = 0.040), and the C allele carriers of rs1993116 had a higher risk of T1D than the T allele carriers, Carriers of the CC and CT genotypes of rs1993116 have higher T1D risk than those carrying the TT genotype, the adjusted ORs (and 95% CI) were 2.411(1.194–4.868) and 2.210(1.115–4.377), respectively. In contrast, there was no significant association between *DHCR7* (rs12785878) and *CYP2R1* (rs12794714) and *CYP24A1* (rs17216707 and *VDR* (rs1544410) genotypes and the risk of T1D.

**Table 3:** Genotyping frequencies of vitamin D-related polymorphisms in T1D and controls.

Polymorphisms		T1D, *n* (%)	Controls, *n* (%)	χ^2^	*p*	Adjusted OR (95% CI)	Adjusted *p* V alue
DHCR7 (rs12785878)				1.143	0.565		
	TT	40 (28.4)	49 (24.5)			1 (Ref)	
Genotypes	GG	33 (23.4)	43 (21.5)			1.006 (0.538–1.883)	0.985
	TG	68 (48.2)	108 (54.0)			0.816 (0.482–1.380)	0.448
Alleles	T	148 (52.5)	206 (51.5)	0.064	0.800		
	G	134 (47.5)	194 (48.5)				
CYP2R1 (rs12794714)				3.997	0.136		
	TT	26 (18.4)	50 (25.0)			1 (Ref)	
Genotypes	CC	53 (37.6)	82 (41.0)			1.212 (0.668–2.199)	0.527
	CT	62 (44.0)	68 (34.0)			1.710 (0.943–3.101)	0.77
Alleles	C	168 (59.6)	232 (36.7)	0.169	0.681		
	T	114 (40.4)	168 (59.6)				
CYP2R1 (rs1993116)				5.682	0.058		
	TT	15 (10.6)	40 (20.2)			1 (Ref)	
Genotypes	CC	57 (40.4)	68 (34.3)			2.411 (1.194–4.868)	0.014
	CT	69 (48.9)	90 (45.5)			2.210 (1.115–4.377)	0.023
Alleles	C	183 (64.9)	226 (57.0)	4.211	0.040		
	T	99 (35.1)	170 (43.0)				
CYP24A1 (rs17216707)				1.373	0.241		
	TT	126 (89.4)	185 (93.0)			1 (Ref)	
Genotypes	CT	15 (10.6)	14 (7.0)			0.645 (0.297–1.399)	0.267
Alleles	C	15 (5.3)	14 (3.5)	1.312	0.252		
	T	267 (94.7)	384 (96.5)				
VDR (rs1544410)				0.571	0.450		
	GG	129 (91.5)	178 (89.0)			1 (Ref)	
Genotypes	GA	12 (8.5)	22 (11.0)			0.753 (0.357–1.588)	0.456
Alleles	G	270 (95.7)	378 (94.5)	0.541	0.462		
	A	12 (4.3)	22 (5.5)				

*n*, number; OR, odds ratio; CI, confidence interval. Logistic regression analysis was performed after adjusting for age and gender.

### SNP-SNP interactions

GMDR was used to evaluate the optimal interaction combination of five SNPs among the 4 candidate genes (Table 4). In general, we found that the two-locus model including rs12794714, and rs1993116 was the best model with statistical significance (cross-validation consistency = 10/10, testing balanced accuracy = 0.6039, *p *= 0.0107), This suggests that there is a potential interaction between rs12794714 and rs1993116 affecting the risk of T1D. However, we did not find significant gene-vitamin D deficiency interactions.

**Table 4:** Gene-gene interaction models in T1D obtained using the GMDR method.

Model	Training Bal. Acc.	Testing Bal. Acc.	CVC	*P* ^a^
Gene-gene interaction				
rs12794714, rs1993116	0.6167	0.6036	10/10	0.0107
rs12794714, rs1993116, rs12785878	0.6451	0.5923	10/10	0.0010
rs12794714, rs1993116, rs12785878, rs17216707	0.6650	0.5624	10/10	0.0547
rs12794714, rs1993116, rs12785878, rs17216707, rs1544410	0.6795	0.5438	10/10	0.0547

Bal. Acc., balanced accuracy; CVC, cross-validation consistency; OR, odds ratio; 95% CI, 95% confidence interval.

^a^ The analyses were performed under logistic regression adjusted for age, gender.

To understand the combined effect of gene-gene interaction on T1D risk, we performed logistic regression analysis on the best model derived from GMDR. The results show that carrying the *CYP2R1* (rs1993116) allele C in conjunction with the *CYP2R1* (rs12794714) genotype CC or allele T increases the risk of T1D, the ORs (and 95% CI) were 2.342(1.218–4.504), 1.956(1.014–3.771), respectively (Table 5).

**Table 5:** Gene-gene interactions influencing T1D based on additive model.

Gene	Gene	T1D	Controls	OR (95%CI)	*P*
rs1993116	rs12794714				
TT	CC	15	39	1 (Ref)	
TT	TT + CT	15	40	0.975 (0.421–2.260)	0.953
CC + CT	CC	38	43	2.298 (1.098–4.807)	0.027
CC + CT	TT + CT	88	117	1.956 (1.014–3.771)	0.045

T1D, type 1 diabetes mellitus; OR, odds ratio; CI, confidence interval.

## Discussion

We designed this case-control study to determine the role of five polymorphisms and their influence on 25(OH)D levels and susceptibility to T1D in Chinese children. we have identified that *CYP2R1* (rs1993116) polymorphism was significantly correlated with T1D in Chinese children. In addition, GMDR analysis showed that there was significant gene-gene interaction among rs12794714 and rs1993116, which was the model with the highest prediction accuracy of the risk of T1D in the five SNPs.

Genome wide association studies (GWAS) meta-analysis of serum 25-hydroxyvitamin D suggests that SNPs in *CYP2R1*, *DHCR7*, *CYP24A1*, and *VDR* genes are associated with circulating 25(OH) D levels (14, 19). However, no evidence of this association was observed in our study. Yan Wang et al. also did not find correlation between *CYP2R1* (rs12794714, rs1993116) and 25(OH) D concentration levels in the Chinese rural population (20). The possible reasons are as follows: (i) There are genetic background and ethnic differences in circulating vitamin D (21), GWAS-related study populations are mostly of European ancestry, and Geographic differences in diet, sun exposure, and genetic background may alter the susceptibility caused by these genetic variants (22). (ii) Each SNP has a small undetectable effect, and combinations of several SNPs may result in an additive effect, leading to a reduction in vitamin D levels. (iii) The sample size of our study is not large enough, and Vitamin D levels are not controlled for seasonal effects.

Several human observational studies have provided evidence for an association between serum 25 (OH) D concentration and the risk of T1D (23, 24), our study also confirmed a higher prevalence of vitamin D deficiency and insufficiency in T1D. As 25(OH)D synthesis is genetically regulated, single nucleotide polymorphisms (SNPs) may alter bioavailability and targeting effects of vitamin D metabolites (25). Previous correlation studies have shown the association of common variant polymorphisms of *DHCR7*, *CYP2R1*, *CYP24A1*, and *VDR* with T1D, providing early support for the causal role of 25(OH) D in the pathogenesis of T1D (8, 26–29). However, some of the results are controversial, Evidence from a Mendelian randomization study and several systematic reviews and meta-analyses suggests an invalid association between selected variants affecting serum 25 (OH) D concentration and T1D (25, 30). The association of *CYP2R1* (rs1993116) with T1D susceptibility in Chinese children was confirmed in our case-control study, We found genotypes “CC” (OR = 2.968, 95% CI = 1.313–6.713, *p *= 0.009,) and “CT” (OR = 2.271, 95% CI = 1.066–4.838, *p *= 0.033,) in *CYP2R1* (rs1993116) is significantly related to an increased T1D risk, Carriers of the CC, CT genotypes of rs1993116 have higher T1D risk than those carrying the TT genotype, and the C allele carriers of rs1993116 had a higher risk of T1D than the T allele carriers.

The *CYP2R1* gene is localized on chromosome 11p15.2 and has five exons, spanning across a region of approximately 15.5 kb, In humans, *CYP2R1* is mainly distributed in the pancreas, liver, and kidney (31). It encodes microsomal vitamin D 25 hydroxylase, which is generally considered to be the most important enzyme in vitamin D metabolism. *CYP2R1* catalyzes the 25 hydroxylation of vitamin D3 and vitamin D2 at comparable rates, In mice, knockout of *CYP2R1* reduced 25(OH) D levels by only 50% and did not affect circulating 1,25(OH)2D (32), Which proves that there is a compensatory mechanism for *CYP2R1* deletion, This may explain the lack of association between *CYP2R1* and 25(OH)D concentration levels in our study. We confirmed that *CYP2R1* (rs1993116) increases the risk of T1D, but *CYP2R1* (rs12794714) does not, The probable reason is that Since rs12794714 is located in an intronic region, these synonymous variants do not alter the protein sequence and therefore have minimal impact on 25-hydroxylase activity and function (33). We originally hypothesized that genetic polymorphisms associated with vitamin D deficiency would increase the risk of T1D, however, our results indicated that *CYP2R1* (rs1993116) was not related to 25(OH)D levels, but was significantly associated with T1D. Whether vitamin D deficiency is a cause or secondary to T1D is still controversial. Due to the relatively limited sample size, these results should be interpreted with caution and further research is required.

Cooper et al. obtained evidence that *DHCR7 and CYP2R1* are associated with T1D (26), A recent study showed that *CYP2R1* (rs12794714) is closely associated with T1D in Korean children (8). However, we did not observe an effective association between *DHCR7* (rs12785878), *CYP24A1* (rs17216707), *CYP2R1* (rs12794714), and *VDR* (rs1544410) polymorphism and T1D in Chinese children, Further research is needed in the future.

Growing evidence suggests that T1D is a polygenic metabolic disease with multiple etiologies, and it is necessary to evaluate gene interactions when studying the genetic etiology of T1D. In our study, we found gene interaction between *CYP2R1* (rs12794714) and *CYP2R1* (rs1993116) was significantly associated with the risk of T1D. Carrying the *CYP2R1* (rs1993116) genotype CC + CT in conjunction with the *CYP2R1* (rs12794714) genotype CC or genotype TT + CT increases the risk of T1D. Although the effect of rs12794714 on T1D is insignificant, the additive interaction between rs12794714 and rs1993116 will increase the risk of T1D.

So far, there are few studies on the relationship between vitamin D, vitamin D-related genes and T1D in Chinese children. We have studied five SNPs of four genes, which can better understand the impact of vitamin D on the pathogenesis of T1D. Also, our study had some limitations, Firstly, the selection bias in this case-control study was its hospital-based design, and it may not be representative of the general population. Secondly, we did not study all the genes involved in the vitamin D metabolic pathway. Finally, our sample size is relatively small, Further expansion of the sample size is required in future studies.

In summary, We studied the triangulation between five SNPs in *CYP2R1*, *DHCR7*, *CYP24A1*, *VDR* genes, serum 25(OH) D levels and T1D in Chinese children. Our study confirms that vitamin D deficiency and insufficiency are more prevalent in T1D, and that *CYP2R1* (rs1993116) polymorphism is a candidate gene for susceptibility to T1D in Chinese children, There was a synergistic effect of gene-gene interactions between *CYP2R1* (rs12794714) and *CYP2R1* (rs1993116) on the risk for T1D, Vitamin D metabolic pathway gene polymorphism, and gene-gene synergy can better explain the effect of vitamin D on the pathogenesis of T1D.

## Data Availability

The original contributions presented in the study are included in the article/**Supplementary Material**, further inquiries can be directed to the corresponding author/s.
