# Supplementary material for: Vitamin D levels and Vitamin D-related gene polymorphisms in Chinese children with type 1 diabetes
Source: Front Pediatr. 2022 Oct 5;10:965296. doi: 10.3389/fped.2022.965296 (PMC9581124; doi:10.3389/fped.2022.965296)
Supplement: Supplementary file 1 [file Table1.docx]

Table S1 Relationship between SNPs genotypes and 3 groups of vitamin D levels (deficient, insufficient and sufficient)

| SNPs | deficiency (＜20ng/mL) | | | Insufficiency (20-30ng/mL) | | | sufficiency (≥30 ng/mL) | | | χ2 | P^a^ |
| --- | --- | --- | --- | --- | --- | --- | --- | --- | --- | --- | --- |
| DHCR7（rs12785878） | GG | TG | TT | GG | TG | TT | GG | TG | TT |  |  |
|  |  |  |  |  |  |  |  |  |  |  |  |
| Total | 33（21.2%） | 83（53.2%） | 40（25.6%） | 31（22.0%） | 73（51.8%） | 37（26.2%） | 12（27.3%） | 20（45.5%） | 12（27.3%） | 1.024 | 0.906 |
| Case | 15(19.0%) | 41(51.9%) | 23(29.1%) | 10(22.2%) | 21(46.7%) | 14(31.1%) | 8(47.1%) | 6(35.3%) | 3(17.6%) | 6.406 | 0.171 |
| Control | 18(23.4%) | 42(54.5%) | 17(22.1%) | 21(21.3%) | 52(54.2%) | 23(24.0%) | 4(14.8%) | 14(51.9%) | 9(33.3%) | 1.777 | 0.777 |
|  | CC | CT | TT | CC | CT | TT | CC | CT | TT |  |  |
| CYP2R1(rs12794714) |  |  |  |  |  |  |  |  |  |  |  |
| Total | 60(38.5%) | 63(40.4%) | 33(21.2%) | 55(39.0%) | 53(37.6%) | 33(23.4%) | 20(45.5%) | 14(31.8%) | 10(22.7%) | 1.296 | 0.862 |
| Case | 27(34.2%) | 36(45.6%) | 16(20.3%) | 20(44.4%) | 17(37.8%) | 8(17.8%) | 6(35.3%) | 9(52.9%) | 2(11.8%) | 2.143 | 0.709 |
| Control | 33(42.9%) | 27(35.1%) | 17(22.1%) | 35(36.5%) | 36(37.5%0 | 25（26.0%）） | 14（51.9%0） | 5(18.5%) | 8(29.6%) | 4.134 | 0.388 |
|  |  |  |  |  |  |  |  |  |  |  |  |
| CYP2R1（rs1993116） | CC | CT | TT | CC | CT | TT | CC | CT | TT |  |  |
| Total | 58(37.2%) | 73(46.8%) | 25(16.0%) | 52(37.4%) | 64(46.0%) | 23(16.5%) | 15(34.1%) | 22(50.0%) | 7(15.9%) | 0.235 | 0.994 |
| Case | 35(44.3%) | 39(49.4%) | 5(6.3%) | 15(33.3%) | 21(46.7%) | 9(20.0%) | 7(41.2%) | 9(52.9%) | 1(5.8%) | 6.410 | 0.171 |
| Control | 23(29.9%) | 34(44.2%) | 20(26.0%) | 37(38.5%) | 45(46.9%) | 14(14.6%) | 8(29.6%) | 13(48.1%) | 6(22.2%) | 4.069 | 0.397 |
|  | TT | CT |  | TT | CT |  | TT | CT |  |  |  |
| CYP24A1rs17216707） |  |  |  |  |  |  |  |  |  |  |  |
| Total | 141(90.4%) | 15(9.6%) |  | 127(90.7%) | 13(9.3%) |  | 43(97.7%) | 1(2.3%) |  | 2.546 | 0.280 |
| Case | 71(89.9%) | 8(10.1%) |  | 39(86.7%) | 6(13.3%) |  | 16(94.1%) | 1(5.9%) |  | 0.770 | 0.680 |
| Control | 70(90.9%) | 7(9.1%) |  | 89(92.7%) | 7(7.3%) |  | 27(100%) | 0 |  | 2.562 | 0.278 |
|  | GG | GA |  | GG | GA |  | GG | GA |  |  |  |
| VDR （rs1544410） |  |  |  |  |  |  |  |  |  |  |  |
| Total | 143(91.7%) | 13(8.3%) |  | 123(87.2%) | 18(12.8%) |  | 41(93.2%) | 3(6.8%) |  | 2.180 | 0.336 |
| Case | 74(93.7%) | 5(6.3%) |  | 39(86.7%) | 6(13.3%) |  | 16(94.1%) | 1 (5.9%) |  | 1.978 | 0.372 |
| Control | 69(89.6%) | 8(10.4%) |  | 84(87.5%) | 12(12.5%) |  | 25(92.6%) | 2(7.4%) |  | 0.606 | 0.739 |

Note: Data are expressed as number (%).

^a^ χ^2^ text.
