# Supplementary material for: Vitamin D levels and Vitamin D-related gene polymorphisms in Chinese children with type 1 diabetes
Source: Front Pediatr. 2022 Oct 5;10:965296. doi: 10.3389/fped.2022.965296 (PMC9581124; doi:10.3389/fped.2022.965296)
Supplement: Supplementary file 2 [file Table2.docx]

Hard-weinberg equilibrium in control group

| *DHCR7*(rs12785878) | GG | TG | TT | χ^2^ | *p* |
| --- | --- | --- | --- | --- | --- |
| observed | 43 | 108 | 49 |  |  |
| expected | 47 | 100 | 53 | 1.311 | **0．252** |
| *CYP2R1 (r*s12794714) | CC | CT | TT |  |  |
| observed | 82 | 68 | 50 |  |  |
| expected | 67 | 98 | 35 | 18.26 | **<0.001** |
| *CYP2R1*（rs1993116） | CC | CT | TT |  |  |
| observed | 68 | 90 | 40 |  |  |
| expected | 65 | 97 | 36 | 1.037 | **0．309** |
| *CYP24A1*(rs17216707) | CT | TT |  |  |  |
| observed | 14 | 185 |  |  |  |
| expected | 13 | 185 |  | 0.264 | **0.607** |
| *VDR* （rs1544410） | GG | GA |  |  |  |
| observed | 178 | 22 |  |  |  |
| expected | 179 | 21 |  | 0.678 | **0.410** |
